# Predictors of Bisexual Behaviour among MSM Attending Intervention Sites May Help in Prevention Interventions for This Bridge to the Heterosexual Epidemic in India: Data from HIV Sentinel Surveillance

**DOI:** 10.1371/journal.pone.0107439

**Published:** 2014-09-11

**Authors:** Sheela Godbole, Suvarna Sane, Pranil Kamble, Yujwal Raj, Nisha Dulhani, Srinivasan Venkatesh, D. C. S. Reddy, Laxmikant Chavan, Madhulekha Bhattacharya, Suchitra Bindoria, Dilip Kadam, Savita Thakur, Prakash Narwani, Elmira Pereira, Ramesh Paranjape, Arun Risbud

**Affiliations:** 1 National AIDS Research Institute (Indian Council of Medical Research), Pune, Maharashtra, India; 2 Department of AIDS Control, Ministry of Health and Family Welfare, Government of India, New Delhi, India; 3 World Health Organisation (India), New Delhi, India; 4 Maharashtra State AIDS Control Society, Mumbai, Maharashtra, India; 5 Mumbai District AIDS Control Society, Mumbai, Maharashtra, India; 6 Madhya Pradesh State AIDS Control Society, Bhopal, Madhya Pradesh, India; 7 Rajasthan State AIDS Control Society, Jaipur, Rajasthan, India; 8 Gujarat State AIDS Control Society, Ahmedabad, Gujarat, India; 9 Goa State AIDS Control Society, Panaji, Goa, India; 10 Department of Community Health Administration, National Institute of Health and Family Welfare, New Delhi, India; University of Athens, Medical School, Greece

## Abstract

**Background:**

Indian cultural tradition demanding marriage, many MSM howsoever they self-identify are likely to be married or have sex with women. To consolidate India's HIV prevention gains, it is important to understand and address the interaction between the MSM and heterosexual epidemics in India and create specific interventions for bisexual MSM. The challenge is to identify and intervene this hard to reach population. Data from HIV Sentinel Surveillance 2011 among MSM in four Indian states were analyzed to assess predictors and prevalence of bisexual behaviour in MSM.

**Methods:**

Between March-May 2011, 4682 men (15–49 years) who had anal/oral sex with a male partner in the past month, attending intervention sites and consenting for an un-linked anonymous survey answered an 11- item questionnaire and provided blood for HIV test by finger stick at 19 designated surveillance sites.

**Results:**

Of 4682 MSM tested overall, 5% were illiterate, 51% reported only receptive anal intercourse, 21% only penetrative and 28% both. 36% MSM had ever received money for sex. Overall 6.8% were HIV infected. 44% MSM were bisexual in the last six months. On multivariate analysis, ‘being bisexual’ was found to be independently associated with ‘older age’: 26–30 years [AOR = 3.1, 95% CI(2.7, 3.7)], >30 years [AOR = 6.5, 95% CI(5.5, 7.7)]; ‘reporting penetrative behaviour alone’ with other men [AOR = 5.8, 95% CI(4.8, 7.0), p<0.01] and ‘reporting both penetrative and receptive behaviour’ [AOR = 2.7, 95% CI(2.3, 3.1) p<0.01]. Those who both paid and received money for sex [AOR = 0.49, 95% CI (0.38, 0.62)] were significantly less likely to be bisexual.

**Conclusions:**

A substantial proportion of men receiving services from Targeted Intervention programs are bisexual and the easy opportunity for intervention in this setting should be capitalised upon. Focusing on older MSM, as well as MSM who show penetrative behaviour with other men, could help in reaching this population.

## Introduction

India has a largely heterosexual HIV epidemic, which is concentrated among various core populations at high risk of HIV [1], [2]. However, Men having Sex with Men (MSM), a recognised core group, are also a powerful driving force [1]–[4]. In 2011, the average national HIV prevalence among MSM was 4.43% and it was greater than 5% in nine Indian states [5]. In contrast however, the estimated adult HIV prevalence among males in general population, who are expected to contribute to the heterosexual epidemic, was much lower (0.32%) [6].

India's National AIDS Control programme (NACP) follows a strategy of targeted programmatic prevention interventions to control the epidemic. MSM are a core population subgroup for these interventions as well as HIV surveillance [1]–[4], [7]. Indian MSM include, self-identified gay men (western acculturated) as well as kothi (receptive male partner), *panthi* or *danga* (penetrative male partner), double-deckers (men with penetrative and receptive behaviours), and *alis* or *hijras* (transgendered people) [3], [8]. HIV transmission risks among MSM vary with the type of sexual behaviour with men [3].

India's NACP recognises MSM as, ‘all men who have sex with other men as a matter of preference or practice regardless of their sexual identity or orientation and irrespective of whether they have sex with women or not’ [3]. However, the ‘Targeted Intervention (TI) program’ which is a key thrust of NACP [4] focuses on ‘high risk MSM’ – i.e. primarily those who are self-identified, practice receptive anal sex and have multiple sexual partners [3].

It is accepted that the prevailing social stigma and legal environment can lead to ‘covert’ behaviour among MSM [9], [10]. It is recognised that many MSM in India do have sex with women (MSMW) [9]. Even though same sex activity is not uncommon in India [11], traditional culture creates pressure to marry. Thus, many MSM irrespective of the way they identify themselves in terms of sexual behaviour or gender are likely to be married [12]. Data from the early years of the Indian epidemic showed that among male STI clinic attendees in Pune (1993–2002), 6.6% reported having sex with men of whom 35.7% were married [13]. Studies from different parts of India with varying sampling techniques have reported that between 11–60% of MSM were married [9], [10], [13]–[17]. Additionally, many authors hypothesize that since ‘married MSM’ are perceived as heterosexuals, they may be isolated from other MSM and therefore less likely to receive prevention intervention in community based settings [14]. Thus they could have more risky sexual encounters with other men, as shown in a study from Chennai [14], [15], [18]. It is also important to note that MSMW have been reported to be less likely to disclose their bisexuality to partners of both sexes.

Although the predominant female partner was the wife (76%), MSM have also reported sex with women outside of marriage including commercial sex workers (13%) [9]. The nationally representative Behavioural Surveillance Survey (2006), revealed a high proportion of MSM reporting sex with a woman in the last six months (30.4%–54.8%) [17]. In the population based Integrated Biological and Behavioural Assessment (IBBA-2006) among MSM in four Indian states, a wide range of MSM reported regular female partners (3.9%–42.7%). The monthly average of 2.1–3.5 female sexual contacts reported by these MSM gives a snapshot of the overlap between heterosexual and same-sex behaviour [16]. A very high HIV prevalence (15.9%) was seen among those who self identified as ‘bisexuals’ in the four state IBBA survey [16].

In a study in Mumbai, married MSM and those with partners of both genders were more likely to be HIV infected [14]. Similarly in south India, the ‘proportion married’ was higher among HIV infected MSM [19].

High rates of STI and HIV and lower rates of condom use, are other factors that add to the potential of men who have sex with men and women in bridging the high HIV prevalent MSM epidemic and the lower-level heterosexual epidemic in general population [9], [10], [13], [16], [17], [20]–[22].

Thus, MSMW in India, with their high HIV prevalence and low condom use with female partners are becoming increasingly recognised as an important bridge population. Their impact on heterosexual HIV transmission dynamics and risk to women who may be married and monogamous needs to be taken into consideration while planning interventions among MSM.

Prevention intervention methods among MSM at ‘Targeted Intervention (TI)’ delivery sites primarily focus on behaviour change communication for safer sex with male partners [4]. Currently interventions focus on ‘high risk MSM’. It is challenging to identify MSMW at these places too and ‘bisexual behaviour’ is difficult to address in such a scenario.

In India, NACP has been conducting frequent and periodic (every one or two years) unlinked anonymous surveillance called HIV Sentinel Surveillance (HSS) since 1998. HSS is designed to measure the level and trends of HIV prevalence in different sub-populations of interest (e.g. MSM, IDU, FSW, ANC attendees) [5], [23]. The most recent HSS among MSM in 2011, collected limited behavioural data along with HIV testing after informed consent, as part of second generation surveillance [5]. This was conducted through organizations providing targeted interventions.

We analyzed data of HSS among MSM in the four Indian states of Goa, Maharashtra (MH), Gujarat, Madhya Pradesh (MP). The specific objectives of our analysis were to identify the prevalence and predictors of bisexual behaviour among MSM attending TI sites and to understand the risk factors for HIV infection among them.

## Material and Methods

### Ethics review and informed consent

The present study was approved by the institutional ethics committee of National AIDS Research Institute, Indian Council of Medical Research (NARI-ICMR).

HSS is a long standing program of National AIDS Control Organization (NACO) -Department of AIDS Control (DAC), Government of India [5], [23]. The Informed consent and assent forms were approved by the ethics committee of DAC and a written informed consent was sought from every respondent.

### Study population

Inclusion and exclusion criteria: MSM attending the DIC of the TI site (MP) or who were listed as current beneficiaries of TI program (Maharashtra, Gujarat and Goa) were eligible for the study. Men who had anal or oral sex with another male (MSM) at least once in the previous month were included in the study. Individuals identifying themselves as *hijra* or transgender were excluded from the study.

Sample size: As per norms of sentinel surveillance in India [5], [23] each site had a sample size of 250 with an anticipated sample of 4750 MSM from 19 sites. It was expected that a minimum 75% (188) sample should be completed in order to use the data of HIV prevalence in HIV estimation process and planning [24]. Based on this we planned to exclude data from study sites which collected fewer than 75% of expected samples.

### Study design

A cross sectional HSS among MSM population was conducted in 19 surveillance sites in the states of Goa (2), Maharashtra (7), Gujarat (8) and Madhya Pradesh (2) in 2011[5]. Standard national technical guidelines were followed by all implementing sites [23]. Selection of surveillance sites was based on epidemiologic need and availability of adequate numbers of the specific target population subgroups. Of the nineteen surveillance sites, surveillance had been conducted more than three times consecutively at 6 sites, between 1 to 3 times at 7 sites while 3 sites in Maharashtra and one each in Goa, Gujarat and Madhya Pradesh were newly initiated in 2011. HSS was conducted under supervision of NARI and the respective State AIDS Control Societies (SACS) which are the state level implementing agencies of the NACP. Trained site in-charges, counsellors and laboratory technicians conducted the actual surveillance at the ‘Drop-in-Centres’ (DIC) established by the organisations providing targeted interventions.

Sampling methods: Two types of strategies viz. random sampling and consecutive sampling were followed during recruitment of respondents in different states.

Random sampling approach was followed in the states of Maharashtra, Goa and Gujarat (17 sites). In order to recruit the sample of 250 MSM the most up to date list of MSM registered as active beneficiaries was obtained and 300 were selected by simple random sampling. The additional 50 numbers were selected to allow for replacements for refusals and inability to contact. The peer educators, who regularly provide services to MSM, received a list of selected MSM from their group. They contacted the selected MSM and invited them to attend the DIC to participate in the surveillance, using a specific standard message. If selected MSM refused to participate or could not be contacted a replacement was taken until sample of 250 was achieved.

At two sites in MP, consecutive sampling was followed. In this method, all eligible MSM who attended DIC during the three-month survey period, were sampled consecutively in the order they attended until sample size was completed [23], [25].

After written informed consent, participants responded to a one page data-form with 11 questions administered by counsellors and provided Dried Blood Spot (DBS) sample for HIV testing. Five drops of capillary blood were collected by finger-stick method using sterile lancet on special protein saver cards. Participants were assigned an ‘HSS ID’ number which was delinked from name and any TI identification number and this number was used as the sole identifier with date for both data forms and blood sample. The respondents did not receive the result of the HIV testing and were referred to the closest government centre for free-of-cost counselling and HIV testing services.

### Study tools

The limited variables of interest collected were: 1) age, 2) education, 3) occupation, 4) current place of residence (rural/urban) and 5) duration of residence, 6) type of sexual practice (kothi, panthi, double-decker, no response), 7) whether had sex with a female in last six months, 8) days since last sex with a male, 9) transactional sex was collected in three mutually exclusive categories: i. ‘received’ or ii. ‘paid’ money (cash/kind) for sex or iii. ‘both received as well as paid’ money for sex and 10) injecting drugs for sake of pleasure in the last one year. Additionally, 11) the reason for coming to DIC was collected only at study sites where consecutive sampling was followed.

During interview, counsellors asked the respondents to classify themselves into the categories of Kothi, Panthi and Double-decker based on their sexual positioning with male partners. They explained that, Kothis refer to receiving partner, Panthi to penetrating partner and Double-decker to those who act as both receiving and penetrating partner during anal sex [25]. Respondents could also refuse to answer this question which was marked no-response.

### HIV Testing and Quality control

A standard two test HIV testing protocol was used for testing DBS. Two ELISA tests, the first test of high sensitivity and the second of high specificity were used. The kits used were (i) Microlisa HIV (J. Mitra & Co. New Delhi) and (ii) Genedia HIV ½ Elisa 3.0 (Green Cross Life Sciences, Ltd. Korea) respectively. Quality control (QC) was done at NARI, Pune by retesting all HIV positive and 2% HIV negative samples.

### Data quality

Regular monitoring and supervisory visits to the TI sites were made by officers from the respective SACS, designated experts identified by NARI and National Institute of Health and Family Welfare during the surveillance. A web-based integrated monitoring and evaluation system was used to monitor quality of survey. This helped us to respond to implementation issues immediately.

Data were entered twice using online data-entry software by two different persons to identify transcription errors in data entry. This software included built-in ‘data matching’ and ‘data monitoring’ functions and ‘validation checks’ which were used for data cleaning and assuring quality. The database that was cleaned and finalized for national HIV estimation was used for this analysis.

### Statistical analysis

For the purpose of the major portion of this analysis we combined data from 19 sites in four states in western and central India in the age group of 18–49 years. This was done under the following assumptions: 1. The identification of predictors of bisexuality & HIV would not be considerably affected by differences in sampling methods (random or consecutive) and 2. Baseline differences in respondents' characteristics between states make no considerable difference in identification of predictors of bisexuality & HIV. However summary statistics for demographic and behavioural characteristics and HIV prevalence data are presented overall and by state. Pearson's Chi-square test was applied to see whether the differences in baseline characteristics are attributed to being from a particular state. This test was also used as the test for association of various characteristics of MSM with bisexual behaviour and HIV infection status. The predicting risk factors and their independent association with bisexual behaviour and HIV was tested using binary logistic regression (forward LR) through univariate and multivariate analysis for combined data of four states. Odds ratios (OR) and adjusted odds ratios (AOR) along with 95% confidence intervals are reported. Z-test for comparison of proportions was used to compare HIV prevalence by bisexuality and sexual behaviour. Results with p value less than 0.05 were considered as statistically significant.

For the purposes of this analysis, men who reported having sex with women in the last six months were classified as MSMW or as having bisexual behaviour while those who did not, were classified as ‘Men having Sex with Men Only’ (MSMO). Type of sexual behaviour with male partner (positioning) was classified as ‘Receptive Only’ for those who reported themselves as kothi, ‘Penetrative Only’ for those who reported themselves as panthi and ‘both’ for those who reported themselves as double-decker.

Since the determinants of bisexuality reported by us were obtained on analysis of combined data from all sites, we performed a sensitivity analysis to understand if state level characteristics or the method of sampling (random versus consecutive) would affect the robustness of findings. We applied the regression model separately for each state, as well as by excluding one state at a time.

## Results

In all 4682 MSM were tested for HIV under sentinel surveillance conducted at 19 sites in 4 states of west-central region of India (Goa, Gujarat, Maharashtra and Madhya Pradesh). All study sites either achieved 100% (n = 12) or over 90% (n = 7) of the expected sample of 250 and were included in analysis.

The mean age of MSM was 28 years (SD = 6.4) which did not substantially differ across states. While 23% had studied beyond matriculation, 5% were illiterate. MSM were predominantly urban residents with about 13% from rural regions. Table 1 shows the state wise differences in characteristics of MSM.

**Table 1:** Demographic and socio-economic characteristics of MSM in states in west-central India (Year 2011).

Characteristic		Four states	Goa	Gujarat	Madhya Pradesh	Maharashtra	P value
No. of MSM respondents (%)		4682	486 (10%)	1968 (42%)	483 (10%)	1745 (37%)	
**Age (in years); Mean (SD)**		28 (6.4)	28 (6.3)	29 (6.7)	27 (6.4)	28 (6.1)	<0.01
**Age group (in years)**	**18**–**25**	1901 (41%)	211 (43%)	744 (38%)	257 (53%)	689 (40%)	<0.01
	**26**–**30**	1452 (31%)	137 (28%)	577 (29%)	120 (25%)	618 (35%)	
	**>30**	1329 (28%)	138 (28%)	647 (33%)	106 (22%)	438 (25%)	
**Education**	**Illiterate (None)**	235 (5%)	22 (5%)	120 (6%)	32 (7%)	61 (4%)	<0.01
	**Till std. 10 (Some)**	3374 (72%)	335 (69%)	1657 (84%)	304 (63%)	1078 (62%)	
	**Beyond std. 10 (Well)**	1069 (23%)	129 (26%)	189 (10%)	145 (30%)	606 (35%)	
Type of MSM*	**Receptive (Kothi)**	2351 (51%)	164 (34%)	1039 (53%)	233 (48%)	915 (53%)	<0.01
	**Penetrative (Panthi)**	970 (21%)	73 (15%)	385 (20%)	46 (10%)	466 (27%)	
	**Both (Double- Decker)**	1318 (28%)	249 (51%)	525 (27%)	203 (42%)	341 (20%)	
	**No response**	28 (0.6%)	0 (0%)	17 (0.9%)	0 (0%)	11 (0.6%)	
Bisexual behaviour (in last 6 months)*	**No (MSMO)**	2590 (56%)	335 (69%)	907 (46%)	364 (75%)	984 (57%)	<0.01
	**Yes (MSMW)**	2060 (44%)	151 (31%)	1051 (54%)	119 (25%)	739 (43%)	
Money exchange for sex*	**No exchange**	2381 (51%)	415 (85%)	855 (44%)	132 (28%)	979 (56%)	<0.01
	**Received (only sell sex)**	1229 (26%)	41 (8%)	623 (32%)	195 (41%)	370 (21%)	
	**Paid (only buy sex)**	605 (13%)	19 (4%)	195 (10%)	70 (15%)	321 (19%)	
	**Both (buy and sell sex)**	445 (10%)	11 (2%)	289 (15%)	83 (17%)	62 (4%)	
History of IDU*	**Yes**	35 (1%)	1 (0.2%)	15 (0.8%)	15 (3%)	4 (0.2%)	<0.01
	**No**	4585 (99%)	482 (99%)	1917 (99%)	463 (97%)	1723 (99%)	
**HIV infected**		332 (6.8%)	22 (4.5%)	59 (3.0%)	39 (7.9%)	173 (9.9%)	<0.01

Note: MSMO: Men having Sex with Men Only, MSMW: Men having Sex with Men and Women, IDU: Injecting Drug Use, *Approx. 0.3%–1.3% data on these variables is missing overall.

About 26% of respondents overall reported being in government or private service while the proportion of MSM who reported working as hotel staff and truck drivers/helpers was 4% and 2% respectively. A considerable proportion of MSM in Madhya Pradesh (29%) reported being students or unemployed which was higher than in other states (student: 2–8%, unemployed: 1–9%). Median duration of stay at current place was reported as 23 years with IQR 15–28. About 483 MSM (10.3%) respondents from two study sites had been sampled consecutively. Among these approximately 10% came to service point to seek medical care including STD treatment while 29% had come to collect condoms and 60% for other reasons which included mostly meeting/event/official work or casually with friend.

Behaviourally 51% reported themselves as receptive only, 21% as penetrative only and 28% as both. The proportion of MSM practising only ‘receptive’ anal sex differed somewhat across other states, the lowest was in Goa (34%). The proportion of MSM practising only penetrative behaviour, was lowest in MP (10%) and highest in MH (27%). Nearly half the respondents had exchanged money for sex and 36% reported only receiving money for sex. Overall 6.8% were HIV infected. MSM who had ever exchanged money for sex had a significantly higher HIV prevalence of 6.6% as compared to 5.9% in those who had never done so (p = 0.02); while prevalence among those who ever sold sex *(only sold or bought and sold)* was 6.3% and those who ever bought sex (*only bought or bought and sold*) was 5.1%.

Forty four percent of MSM reported having sex with women in last 6 months and were classified as MSMW. Prevalence of HIV among MSMW was 5.3%. Anal sex positioning with male partners among MSMW and MSMO were similar. Maximum MSMW reported receptive only (35.6%), while only penetrators formed 30.9% and both types were 33.5%.

On multivariate analysis, MSMW were found to be independently associated with being older; 26–30 years [AOR = 3.1, 95% CI (2.7, 3.7)], >30 years [AOR = 6.5, 95% CI (5.5, 7.7)]. MSM who reported penetrative behaviour alone with other men were nearly six times more likely to be MSMW [AOR = 5.8, 95% CI (4.8, 7.0), p<0.01]; while MSM reporting both penetrative and receptive behaviour were nearly thrice as likely to have sex with women [AOR = 2.7, 95% CI (2.3, 3.1), p<0.01]. Those who both ‘paid’ and ‘received’ money for sex [AOR = 0.49, 95% CI (0.38, 0.62)] were significantly less likely to be bisexual (Table 2).

**Table 2:** Predictors of bisexual behaviour among MSM in west-central India (Year 2011).

Characteristic		All MSM (%), n = 4650*	MSMW (%), n = 2060	OR (95% CI)	P value	AOR (95% CI)	P value
**Age group (in years)**	**18**–**25**	1895 (41%)	536 (28%)	Referent		Referent	
	**26**–**30**	1438 (31%)	695 (48%)	2.4 (2.1, 2.7)	<0.01	3.1 (2.7, 3.7)	<0.01
	**>30**	1317 (28%)	829 (63%)	4.3 (3.7, 5.0)	<0.01	6.5 (5.5, 7.7)	<0.01
**MSM Behaviour**	**Receptive**	2334/4615 (51%)	729 (31%)	Referent		Referent	
	**Penetrative**	970/4615 (21%)	633 (65%)	4.1 (3.5, 4.8)	<0.01	5.8 (4.8, 7.0)	<0.01
	**Both**	1311/4615 (28%)	686 (52%)	2.4 (2.1, 2.8)	<0.01	2.7 (2.3, 3.1)	<0.01
Money exchange for sex*	**No exchange**	2372/4636 (51%)	1118 (47%)	Referent		Referent	
	**Received**	1216/4636 (26%)	430 (35%)	0.61 (0.53, 0.71)	<0.01	0.88 (0.75, 1.03)	0.114
	**Paid**	604/4636 (13%)	368 (61%)	1.75 (1.46, 2.1)	<0.01	1.2 (0.95, 1.5)	0.128
	**Received+Paid**	444/4636 (10%)	135 (30%)	0.49 (0.39, 0.61)	<0.01	0.49 (0.38, 0.62)	<0.01
**History of IDU**	**No**	4562/4597 (99.2%)	2010 (44%)	Referent		Referent	
	**Yes**	35/4597 (0.8%)	23 (66%)	2.4 (1.2, 4.9)	0.013	2.1 (1.0, 4.6)	0.05

Note: Column 2: Denominators for percentages are respective figures in 1st column. OR: Odds Ratio; AOR: Adjusted Odds Ratio. * Missing information about bisexuality for 32 records; IDU: Injecting Drug Use, MSMW: Men having Sex with Men and Women. * ‘Received Money’ =  Only sold sex, ‘Paid’ = Only bought sex; ‘Received+Paid’ =  Bought and sold sex.

Multivariate analysis showed that paying for sex [paid money: AOR = 1.5, 95% CI (1.03, 2.2)]; receptive MSM behaviour [AOR = 1.8, 95% CI (1.2, 2.7)] and older age; 26–30 years, [AOR = 1.5, 95% CI (1.1, 2.0)], >30 years [AOR = 1.9, 95% CI (1.4, 2.6)] showed independent association with HIV infection (Table 3).

**Table 3:** Predictors of HIV infection among MSM in west-central India (Year 2011).

**Characteristic**		**All MSM (%); n = 4682**	**HIV infected (%); n = 292**	**OR (95% CI)**	**P value**	**AOR (95% CI)**	**P value**
**Age (in years)**	**18**–**25**	1901 (41%)	92 (4.8%)	Referent			
	**26**–**30**	1452 (31%)	97 (6.7%)	1.4 (1.05, 1.9)	0.023	1.5 (1.1, 2.0)	0.011
	**>30**	1329 (28%)	103 (7.8%)	1.7 (1.2, 2.2)	0.001	1.9 (1.4, 2.6)	<0.01
**MSM Behaviour**	**Penetrative**	970/4639 (21%)	42 (4.3%)	Referent		Referent	
	**Receptive**	2351/4639 (51%)	188 (8.0%)	1.9 (1.4, 2.7)	<0.01	1.8 (1.2, 2.7)	<0.01
	**Both**	1318/4639 (28%)	59 (4.5%)	1.04 (0.7, 1.6)	0.866	1.1 (0.7, 1.7)	0.669
**Bisexual behaviour (in last 6 months)**	**No**	2590/4650 (56%)	181 (7.0%)	Referent			
	**Yes**	2060/4650 (44%)	110 (5.3%)	0.75 (0.59, 0.96)	0.022	0.7 (0.5, 0.9)	0.015
Money exchange for sex*	**No exchange**	2381/4660 (51%)	141 (5.9%)	Referent			
	**Received**	1229/4660 (26%)	96 (7.8%)	1.3 (1.03, 1.8)	0.030	1.1 (0.9, 1.5)	0.390
	**Paid**	605/4660 (13%)	44 (7.3%)	1.2 (0.9, 1.8)	0.219	1.5 (1.03, 2.2)	0.033
	**Received+Paid**	445/4660 (10%)	10 (2.2%)	0.4 (0.2, 0.7)	0.002	0.32 (0.17, 0.62)	0.001
**History of IDU**	**No**	4692/4620 (99%)	288 (6.3%)	Referent			
	**Yes**	91/4620 (1%)	2 (5.7%)	0.9 (0.2, 3.8)	0.890	—	—

Note: Column 2: Denominators for percentages are respective figures in 1st column. OR: Odds Ratio; AOR: Adjusted Odds Ratio; IDU: Injecting Drug Use. * ‘Received Money’ = Only sold sex, ‘Paid’ = Only bought sex; ‘Received+Paid’ =  Bought and sold sex.

### Results of sensitivity analysis

#### Sensitivity analysis for assumption 1

The results of analysis of 3 states (Gujarat, Maharashtra and Goa), where random sampling methodology was executed, did not show any remarkable difference in independently associated predictors of bisexuality and HIV infection as compared to the results of combined analysis of all 4 states.

#### Sensitivity analysis for assumption 2

While analyzing association of the above mentioned variables with bisexuality, all the states demonstrated the independent association of older age group with bisexuality (adjusted odds ratios ranging from 2.4 to 11.5). MSM with penetrative behaviour were more likely to be bisexual than those with receptive behaviour, in all the states (AOR from 5.0 to 15.5). Additionally, MP showed an association of bisexuality with money exchange for sex [AOR = 2.0, 95% CI (1.2, 3.3); p = 0.008] and history of injecting drug use [AOR = 4.9, 95% CI (1.5, 16.7); p = 0.010].

Sensitivity analysis shows that, the predictors of bisexuality are not sensitive to method of sampling or geographic location i.e. state.

When we performed separate state wise analysis, none of the behavioural or demographic factors were significantly associated with HIV infection in the states of Madhya Pradesh and Goa. In Gujarat & Maharashtra, receptive MSM were more likely to be HIV infected as compared to penetrative MSM [GJ: AOR = 4.3, 95% CI (1.5, 12.1); p = 0.005] & [MH: AOR = 1.9, 95% CI (1.3, 2.9); p = 0.003]. Additionally in the state of Maharashtra older age was independently associated with being HIV infected [26–30 years: AOR = 1.7, 95% CI (1.1, 2.5), p = 0.013 and >30 years: AOR = 2.0, 95% CI (1.3, 3.0), p = 0.001] as compared to 18–25 years age group.

## Discussion

In our analysis of HSS data from 4682 MSM sampled in western and central India, we highlight a high proportion of bisexual behaviour and identify the key determinants for the same. Our findings also emphasize the importance of transactional sex and HIV prevalence in this population. India's HIV program utilizes programmatic data for improvement of services and prevention efforts. Our findings, can guide prevention programming for MSM in India's recently initiated NACP Phase IV [4], [26].

Close to a half (44%) of the MSM attending TI sites have sex with women as well. The HIV prevalence in this population (5.3%) is higher than the overall national prevalence for MSM and is of concern due to transmission risks to men as well as women partners. Of importance, is our finding that, MSM who practice penetrative behaviour with other men, are nearly six times more likely to have sex with women, as compared to *Kothi* (AOR 5.8). Practicing both penetrative and receptive behaviour with men, also emerged as being significantly predictive of bisexual behaviour (AOR 2.7). That our data highlight penetrative behaviour with other men, as a very significant predictor for bisexual behaviour is probably not entirely unexpected. However, of note is the finding that this remains significant even when men also practice receptive behaviour with other men.

The study also highlights that increasing age was significantly associated with having sex with women. MSM older than 30 years, were six and half times more likely and MSM between 25–30 years of age nearly thrice as likely to be behaviourally bisexual, as compared to MSM below 25 years. A recent analysis from south India which reported that ‘typically more insertive MSM are older’ has provided context to other anecdotal reports that sexual behaviours do change over time and with increasing age [27]. Our data from other parts of India (western and central) too support these reports. This therefore raises further questions about determinants and origins of ‘change in behaviour to being bisexual’ among older MSM. We suggest that these questions should be a focus for future qualitative research in the Indian context.

In contrast to the majority of Indian studies which focused on ‘Married MSM’ [7], [13], [14], our study focuses on behaviourally bisexual men (44% MSMW). The proportions are similar to the south-Indian study (2003–04), which showed that behaviourally bisexual MSM were more than those currently married (41%); highlighting that MSM need not be married to have sex with other women [9], [16], [20].

There is a slowly increasing recognition of the importance of this subset of the MSM population for India's HIV epidemic. However, the main challenge has been ‘how to identify behaviourally bisexual men in the community for prevention interventions?’ [15], [19]. Our study highlights that, the high proportion of MSMW already attending targeted intervention programs are a prevention opportunity, which should not be missed. Findings on determinants of bisexual behaviour obtained from this study will help as a starting point to identify MSM with female partners. In this context, we caution that this needs to be accompanied by development of focused and appropriate behaviour change communication messaging, addressing the bridging potential of MSMW. Current guidelines for ‘Peer Educators’, which do not include specific counseling messages about ‘risk to female partners’, ‘safer anal/vaginal sex with females’ and ‘disclosure to and communication with females’ may thus require enhancement to address this prevention need [28].

Our study also found a higher HIV prevalence in MSM population in all four states in the west-central region than the national average [5]. This is higher than the prevalence in the core group for heterosexual epidemic i.e. FSW. We would also like to highlight that MSMW were highly represented in the states of Maharashtra (43%) and Gujarat (54%). In these two states, the HIV prevalence among women attending antenatal clinics is higher than national average and these states would be useful models to study the intersection of the MSM and heterosexual epidemics in India. Additionally in this study we report data about MSMW from low HIV prevalence state of Madhya Pradesh (25%) for the first time which will be useful for modeling and estimation purposes.

This high prevalence of both HIV and bisexual behaviour thus adds further emphasis to prevailing data [21] that this core risk group also functions as a bridging population. Addressing this bridging potential is of immense importance if the current successes in India's prevention programming are to be maintained.

In this study, individuals identifying themselves as *hijra* or transgender who are traditionally likely to undertake sex work were excluded. In spite of this, transactional sex was very common with nearly half the respondents ever having exchanged money for sex, while about a third (36%) had ever received money for sex. This group showed much higher prevalence and risk of HIV infection. We also found that MSM who both ‘paid’ and ‘received’ money for sex were less likely to be bisexual. A possible explanation for this could be that these men may engage primarily in transactional sex and therefore are most likely to preferentially practice receptive sex.

These data suggest that strategies specifically addressing the socio-economic contextual factors surrounding transactional sex among MSM should be a key focal point in NACP IV. Formative research to tease out the underlying constructs of transactional sex for development of interventions would be productive.

Our analysis of sentinel surveillance data does have some limitations. The surveillance occurred at TI service points, which included active MSM visible in the hot spots. Since they were recruited at a time when the programmatic focus was on ‘higher-risk’ MSM, they may not be entirely representative of the whole population. Respondents had already been coming to the TI sites and they self-identified themselves by behavioural typology (kothi, panthi or double-decker) which were mutually exclusive. This however may not be representative of the entire MSM population in their regions especially the hidden population or those who are not self-identified MSM. To reach hidden MSM, sampling strategies like respondent driven sampling can be implemented in the future and may even be considered for enhancing recruitment and registration at TI sites [29]–[31].

Although there was some difference in the sampling strategy between states, majority were sampled by random method. Sensitivity analysis by sampling methods did not show any remarkable difference in the results. This strengthens our assumption that sampling method may not have introduced significant bias for the purpose of this analysis for predictors of bisexual behaviour. Similarly analysis by individual state or by eliminating one state at a time from the model has proved the robustness of results.

## Conclusions

In light of the 17.7 million sexually active males who are estimated to be same-sex oriented and engaging in same sex behaviour in India [26], our findings highlight the importance of focusing interventions in a subpopulation that is more likely to act as bridge to the low or no-risk women in the general population.

Our study shows, that a substantial proportion of ‘high risk’ MSM who receive services from TI sites have sex with men as well as women and the easy opportunity for intervention in this group should be capitalized upon. We emphasize, that these findings will be useful to the program which is largely TI based. Predictors of bisexuality identified in this analysis will help to devise prevention interventions focused on MSMW within the existing targeted intervention programs. We highlight that an additional approach could be to ensure that these prevention messages addressing protection of partners of any gender as an intervention are also targeted towards self- identified MSM who are older as well as show penetrative behaviour with other men especially those already attending TI sites.
